# Fibrosis: a key feature of Fabry disease with potential therapeutic implications

**DOI:** 10.1186/1750-1172-8-116

**Published:** 2013-08-06

**Authors:** Frank Weidemann, Maria D Sanchez-Niño, Juan Politei, João-Paulo Oliveira, Christoph Wanner, David G Warnock, Alberto Ortiz

**Affiliations:** 1 Department of Medicine, Divisions of Cardiology and Nephrology, The Comprehensive Heart Failure Center at the University of Würzburg, Würzburg, Germany; 2 IDIPAZ/REDINREN, Madrid, Spain; 3 Trinity Dupuytren Clinic, Neurology department, Buenos Aires, Argentina; 4 Centro Hospitalar de São João, Porto, Portugal; 5 University of Alabama at Birmingham, Birmingham, AL, USA; 6 IIS-Fundacion Jimenez Diaz-UAM, IRSIN/REDINREN, Madrid, Spain; 7 Unidad de Dialisis, IIS-Fundacion Jimenez Diaz, Av Reyes católicos 2, Madrid, 28040, Spain

**Keywords:** Fabry, Fibrosis, Podocyte, Lyso-Gb3, Kidney, Heart, Enzyme replacement therapy

## Abstract

Fabry disease is a rare X-linked hereditary disease caused by mutations in the AGAL gene encoding the lysosomal enzyme alpha-galactosidase A. Enzyme replacement therapy (ERT) is the current cornerstone of Fabry disease management. Involvement of kidney, heart and the central nervous system shortens life span, and fibrosis of these organs is a hallmark of the disease. Fibrosis was initially thought to result from tissue ischemia secondary to endothelial accumulation of glycosphingolipids in the microvasculature. However, despite ready clearance of endothelial deposits, ERT is less effective in patients who have already developed fibrosis. Several potential explanations of this clinical observation may impact on the future management of Fabry disease. Alternative molecular pathways linking glycosphingolipids and fibrosis may be operative; tissue injury may recruit secondary molecular mediators of fibrosis that are unresponsive to ERT, or fibrosis may represent irreversible tissue injury that limits the therapeutic response to ERT. We provide an overview of Fabry disease, with a focus on the assessment of fibrosis, the clinical consequences of fibrosis, and recent advances in understanding the cellular and molecular mechanisms of fibrosis that may suggest novel therapeutic approaches to Fabry disease.

## Fabry disease

Fabry disease is a rare X-linked hereditary disease caused by mutations in the *GLA* gene encoding the lysosomal enzyme alpha-galactosidase [1]. Males with classical disease are severely affected, while in females the random inactivation of one X chromosome underlies a wide spectrum of severity [2]. Disease manifestations are a consequence of the accumulation of glycosphingolipids in lysosomes and extralysosomal and extracellular spaces [3]. However, the precise cellular and molecular mechanisms linking glycolipid accumulation to tissue injury and disease manifestation are not fully understood. The lack of an adequate animal model for the disease has hindered progress in understanding the pathogenesis and, development of optimal therapy.

**Figure 1:**
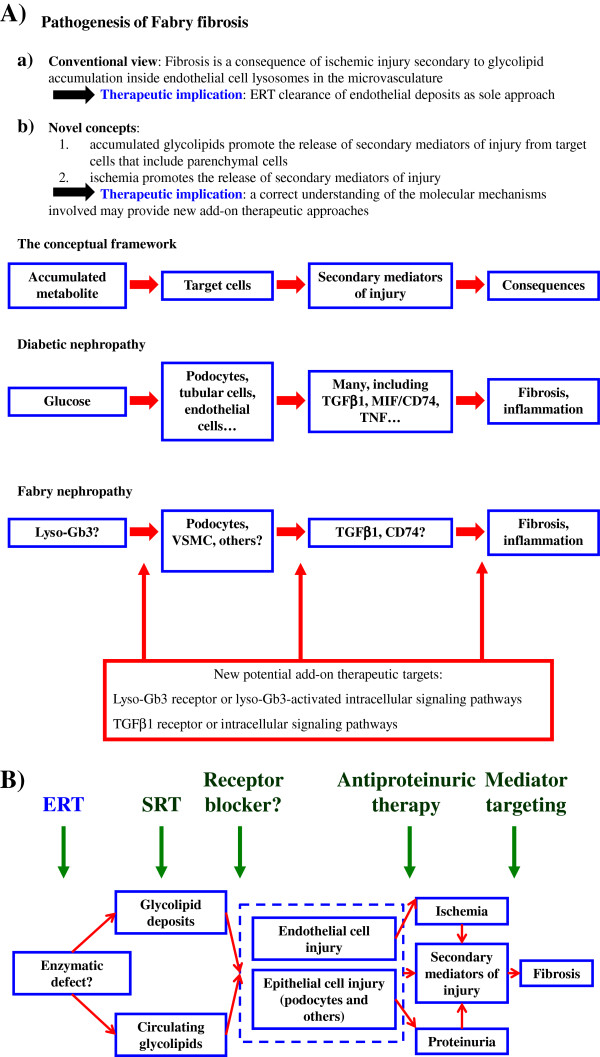
**Conceptual framework for the design of novel therapeutic approaches to Fabry disease: lessons from diabetic nephropathy. A)** Pathogenesis of Fabry fibrosis. The traditional view is that this is a late event secondary to endothelial glycolipid deposition leading to luminal obstruction and ischemia. However, fibrosis in other metabolic disorders, such as diabetes, is known to result from recruitment of secondary mediators of injury by both direct actions of accumulated metabolites (in this case glucose) on target organ cells and also by ischemia. Recent evidence suggests that certain metabolites that accumulate in Fabry disease may recruit secondary mediators of injury in target organ cells. Such pathways might be amenable to therapeutic targeting by preventing the effects of accumulated metabolites on target cell or by targeting the secondary mediators that are recruited. **B)** Potential impact on therapy of an improved understanding of the pathogenesis of fibrosis in Fabry disease. Current therapy of Fabry disease consists of enzyme replacement therapy (ERT). Substrate reduction therapy (SRT) in under investigation and may further decrease the levels of certain metabolites identified as pro-fibrotic. Identification of metabolites recruiting secondary mediators of injury may eventually lead to therapies preventing their binding to receptors. In addition, anti-proteinuric therapy may decrease the pro-inflammatory, pro-fibrotic effects of proteinuria in the kidney. Certain anti-proteinuric agents have additional anti-fibrotic actions in the kidney and vasculature. Finally, targeting of secondary mediators of fibrosis may further prevent fibrosis progression in patients with more advanced disease for whom correction of the initial metabolic defect may not be sufficient.

Initial symptoms of Fabry disease usually appear in childhood and reduce the quality of life but are not life-threatening [1]. These include angiokeratoma, neuropathic pain, hypohydrosis and digestive tract symptoms. During the second decade of life, potentially life-threatening involvement may develop, including the central nervous system (CNS), including stroke, chronic kidney disease (CKD) usually associated with proteinuria and progressive loss of glomerular filtration rate (GFR), and left ventricular (LV) hypertrophy, arrhythmia and heart failure. Fibrosis of these organs is a key feature of Fabry disease. Enzyme replacement therapy (ERT) is the current cornerstone of Fabry disease management [1,4,5] (Figure 1). ERT is less efficacious when started after the development of tissue injury and specifically, of tissue fibrosis [6]. ERT should be complemented by symptomatic therapy and by adjuvant therapy aimed at modifying the underlying pathogenic mechanisms of tissue injury, such as targeting the renin-angiotensin-aldosterone system (RAAS) to reduce proteinuria [4,5,7]. Novel therapeutic approaches based on a better understanding of pathogenic events are needed to complement ERT and optimize patient outcomes. In this review we discuss the current understanding of fibrosis in Fabry disease, and address following questions: What is the contribution of fibrosis to disease burden in Fabry disease? What are the cellular and molecular mechanisms of fibrosis? How can fibrosis be assessed? And what are the prospects for fibrosis-guided therapy?

## Fibrosis as a feature of organ damage

Fibrosis is characterized by an increased accumulation of extracellular matrix (ECM) [8-11]. Fibrosis or the formation of scar tissue can be the end-result of tissue injury, inflammation and apoptosis and might be considered a final irreversible event with little intrinsic therapeutic interest [8-11]. However, in some clinical conditions fibrosis is an early event, a disease defining-event or a major contributor to clinical manifestations of disease. There is evidence that diabetic nephropathy (DN) and Fabry disease may be such conditions. Like DN, Fabry nephropathy is a proteinuric nephropathy of metabolic origin characterized by a progressive decrease of renal function to a terminal stage requiring dialysis or transplantation. Although the metabolic environments of the two diseases are considerably different, there is accumulating evidence that they may share common, later-stage pathogenic pathways with other forms of proteinuric CKD. Advances in the understanding of fibrosis regulation in prevalent diseases, such as DN, and their therapeutic implications may be used to develop therapeutic approaches to less common conditions like Fabry disease. In DN intrinsic renal cells are early contributors to kidney fibrosis. Thus, initial glomerular (GBM) and tubular basement membrane thickening depends on increased production of ECM by glomerular epithelial podocytes and tubular epithelial cells injured by high ambient glucose concentrations [12-15]. This is followed by recruitment of activated fibroblasts, focal and segmental glomerular fibrosis and sclerosis (FSGS) and interstitial fibrosis. Interestingly, ECM undergoes remodeling and ECM deposits are potentially reversible. Following pancreas transplantation in patients with DN, increased ECM deposition of metabolic origin is reversible following 10 years of continuous correction of the metabolic defect, but not after 5 years [16]. ERT provides clinically significant, but not complete reversal of the Fabry metabolic defect. Glycolipid deposits may persist for years in certain cell types, such as podocytes, the key cells in glomerulosclerosis and proteinuria [6,17] and circulating levels of deacylated globotriaosylceramide (globotriaosylsphingosine, lyso-Gb3) are reduced but not normalized by ERT [18-20]. Depending on dose [17], ERT provides partial control of the metabolic defect in a manner similar to oral anti-diabetic agents and insulin in diabetes than to the cure offered by pancreas transplantation. ERT may be less effective in controlling the metabolic defect due to pre-existent deposits, sub-optimal dose, and antibodies or due to poor tissue penetration. Similarly, diabetic patients treated with antidiabetic medications still require adjuvant, tissue-protective therapies, and a similar paradigm applies to Fabry disease (Figure 1). Furthermore, any potential beneficial effect of ERT to ameliorate or reverse fibrosis is expected to take many years, especially if fibrosis is well established before ERT is started.

## Fibrosis in Fabry disease

**Figure 2:**
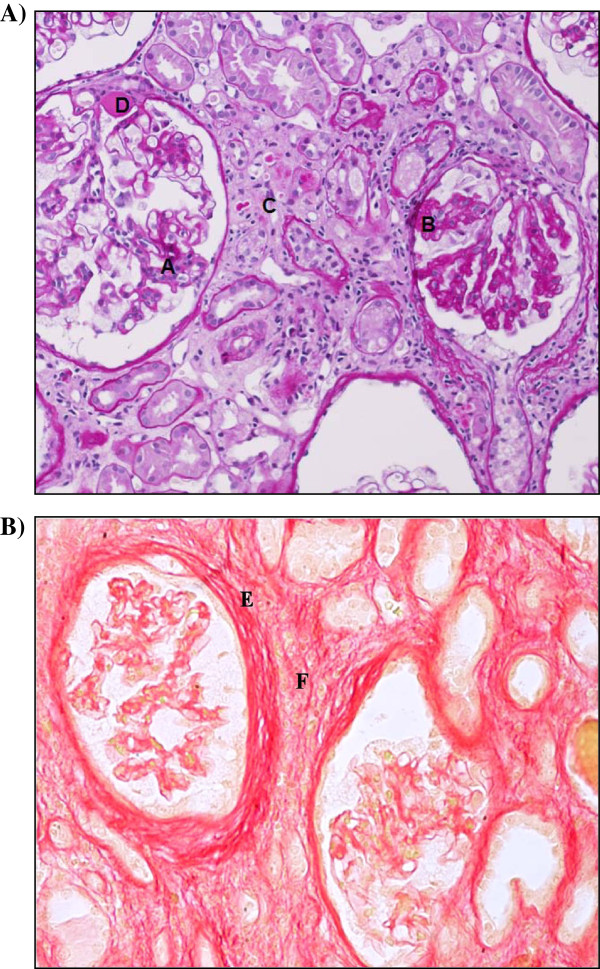
**Kidney biopsy. A)** PAS staining. Histology of the kidney with characteristic changes of advanced Fabry nephropathy. Please note glomerular segmental sclerosis **(A)**, adhesion and Bowman capsule reduplication **(B)**, tubular atrophy and tubular cell related fibrosis (thickened basement membranes) **(C)** and arteriolar hyalinosis **(D)**. Original magnification 63×. Courtesy of Prof. Justus Müller, Department of Pathology, University Hospital of Würzburg, Würzburg, Germany. **B)** Sirius red staining of collagen fibers illustrates peri-glomerular fibrosis **(E)** and interstitial fibrosis **(F)**. Original magnification × 20.

Fibrosis can be found in histological sections of Fabry disease targets organs. Renal fibrosis is a feature of Fabry nephropathy. The time-course of kidney fibrosis is not as clearly established as in DN, but emerging evidence points to a similar pattern: early podocyte injury and fibrosis generated by epithelial cells that increase as disease progresses [17,21-23] (Figure 2). A grossly thickened GBM was noted in early reports of Fabry nephropathy and GBM duplications and increased glomerular mesangial ECM are also found [21,24-27]. Glomerulosclerosis and interstitial fibrosis are already present in children with early stage tissue injury characterized by preserved renal function and albuminuria <300 mg/g creatinine, along with features of podocyte injury such as segmental foot-process effacement [17,21]. Glomerular sclerosis and interstitial fibrosis may also be observed in females with normal renal function and in the absence of overt proteinuria [23]. In a cross-sectional study of 59 male and female Fabry patients the mean percentage of non-sclerosed glomeruli was 82±19% in 25 patients with well preserved renal function (mean estimated GFR = 113 ml/min/1.73 m^2^) and 21±14% in 5 patients with severe CKD (eGFR= 16 ml/min/1.73 m^2^). Mean percentage of interstitial fibrosis area was 8±16% and 66±14%, respectively [22].

The typical clinical presentation of Fabry cardiomyopathy is LV hypertrophy. Most patients with a cardiomyopathy exhibit a concentric LV hypertrophy with an end-diastolic wall thickness of up to 16 mm without concomitant LV outflow tract obstruction [28]. Typical features of Fabry cardiomyopathy include prominence of the papillary muscle [29-31] and development of replacement fibrosis in the basal postero-lateral segments [32-34]. In addition, biopsies have shown interstitial fibrosis at early cardiomyopathy stages. The fibrotic process starts in the mid-myocardial layers and spreads with disease progression towards transmural fibrosis. Thus, the end-stage of the cardiomyopathy is characterized by the co-existence of LV hypertrophy, myocardial thinning, and the presence of wall motion abnormalities in the fibrotic segments [35,36].

In female Fabry patients, LV hypertrophy and fibrosis seems to be not tightly linked [37], perhaps reflecting the residual alpha galactosidase A activity in females. Replacement fibrosis can already be present at a non-hypertrophic disease stage, which is in contrast to males who normally first develop LV hypertrophy and subsequent replacement fibrosis. In addition, in all female patients who develop LV hypertrophy, replacement fibrosis is present. Thus, despite the delayed development of LV hypertrophy, fibrosis seems to progress continuously and is an integral component of the cardiomyopathy [37] in female patients with Fabry disease.

Much less is known about fibrosis of the CNS in Fabry disease. Indeed the general pathogenesis of fibrosis in the CNS is poorly understood. The term gliosis or glial reaction indicates structural and physiological changes of astrocytes and microglia in response to ischemic, inflammatory or traumatic injuries to the CNS. A prominent feature of gliosis is the proliferative response to injury that is followed later by permanent changes, the glial scar [38]. Gliosis is known to occur in the penumbra area adjacent to the ischemic core during stroke [39]. In Fabry disease gliosis and fibrosis have been reported at sites of stroke [25]. Pathological findings secondary to ischemic encephalopathy included fibrillar astrocytosis and proliferation of microglia adjacent to pyknotic neurons in the hippocampus, cerebral cortex and white matter [40]. In addition, features of a more generalized fibrotic process were also observed in Fabry disease, such as thickening of the pia-arachnoid membranes and an angiopathy of the subarachnoidal arteries characterized by intima and medial thickening and fibrosis and adventitial fibrosis associated with gliosis [25,41].

## Pathogenesis of fibrosis in Fabry disease

Improved understanding of fibrosis in Fabry disease will permit development of more effective therapeutic approaches to Fabry disease. “Fabry mice” (GLA −/− and -/0 mice) display mild accumulation of glycosphingolipids but have thus far failed to develop significant kidney or heart disease [42]. The lack of significant end-organ fibrosis may be due to a lower accumulation of glycosphingolipids in mice, differences in lipid metabolism between mice and humans, the potential need for several years of progressive glycolipid accumulation and the genetic background of current Fabry mouse models. In the absence of an adequate animal model only hypothesis based on human histology or cell culture models are available. Previous reports emphasized that the histological appearance of advanced Fabry nephropathy suggested that kidney fibrosis was a consequence of ischemia [24,26]. Thus, in 25 to 50 year old patients, glomerulosclerosis, often with wrinkled and partially collapsed GBM, tubular atrophy and interstitial fibrosis were thought to result from the also present vascular thickening. However, these are non-specific features of advanced kidney disease of any etiology. Moreover, these changes were generally minimal in patients <25 years, further supporting the notion that these are secondary features of the disease and that early pathogenic events may differ. At that time the key importance of podocytes in the maintenance of the glomerular filtration barrier to proteins and the pathogenesis of glomerulosclerosis was unknown. It is now widely accepted that podocyte injury is a key event in the development of proteinuric kidney disease, and that podocyte loss is the main driver of glomerulosclerosis [12]. More recently it has been recognized that podocytes are among the earliest cells to be loaded with glycolipid deposits. Moreover, podocyte deposits volume density increased progressively with age (unlike endothelial or mesangial inclusion volume densities). Foot process width was greater in male Fabry patients and progressively increased with age compared with the controls, and correlated directly with proteinuria [43]. Finally, podocyte effacement, a manifestation of podocyte injury, is observed in children with minimal albuminuria [21]. Hence, podocyte injury has been proposed to play a pivotal role in the development and progression of Fabry nephropathy [43]. In this regard, podocyte deposits are the less responsive to ERT in adults and may take up to 5 years of continued ERT to show significant clearing [6,17,44]. The clinical correlate is lack of improvement of proteinuria by ERT in adults. By contrast, kidney endothelial cells and fibroblasts deposits are cleared within 6–12 months of 1 mg/kg/2 weeks ERT [44]. More support for the concept of a key role of podocytes in Fabry nephropathy and the need of clearance of podocyte deposits comes from the observation that in young patients podocytes can be cleared by several years of ERT at 1 mg/kg/2 weeks, and this was associated with regression of “micro-albuminuria” [17]. In addition to potential direct effects of glycolipids on tubular cells, proteinuria itself may lead to tubular cell activation, inflammatory responses and interstitial fibrosis.

In cardiomyocytes, GL-3 storage, trophic factors and other factors (e.g. lyso-Gb3) and ischemia at the microcirculatory level are supposed to directly cause injury and alter the expression of signaling molecules [18,45,46], triggering inflammation, hypertrophy, apoptosis, increased deposition of extracellular matrix (early interstitial fibrosis), and late cell-replacement fibrosis [33,47,48]. The elevated lyso-Gb3 in plasma of symptomatic patients might partially explain the finding by Barbey *et al.*[49] of an unidentified substance in plasma of symptomatic Fabry disease patients that stimulates proliferation of vascular smooth muscle cells and cardiomyocytes *in vitro*. Of note, a correlation was observed between left ventricular hypertrophy and plasma lyso-Gb3 concentration in heterozygote Fabry patients [18]. In addition, it was speculated that wall stress may contribute to Fabry cardiomyopathy. Thus, slightly increased blood pressure and the flat curvature of the basal part of the lateral wall may account for an increased wall stress that promotes fibrosis. This scenario would account for fibrosis that starts in the endocardium (where wall stress is highest) but not at the mid-myocardium as is seen in the cardiac involvement in Fabry disease [50]. Thus, other unknown factors are contributing to hypertrophy and fibrosis. The relative contribution of cardiomyocytes versus other cell types to myocardial fibrosis in Fabry disease is unclear.

It has long been thought that CNS gliosis in Fabry results from ischemia. The anatomical location of white matter lesions also supports an ischemic origin, although the mechanisms of ischemia remain unclear [51,52]. Brain white matter is localized below the cortex and contains axons from neurons. White matter lesions are defined by the presence of bright spots (T2 and FLAIR sequences) in this region in brain imaging. Perfusion of the white matter depends on the long penetrating arteries originating from the cortical surface that are perpendicular to the cortex and follow the course of myelinated fibers. Because there are minimal or no anastomoses between sub-ependimal vessels and vessels originating from the cortex, watershed periventricular areas are susceptible to ischemic injury from decreased cerebral blood flow [53]. However, the decreased blood flow does not result from the involvement of intracerebral vessels by the glycolipid storage process, and other (as yet unidentified) hemodynamic factors might also be involved. Khan described massive dilatation of the vertebral and basilar arteries in autopsies, but absence of glycolipid deposits in intracerebral vessels, despite the marked thickening of the media of small arteries and arterioles caused by deposition of the glycolipid observed in almost every tissue, including the leptomeninges of the brainstem and spinal cord [25]. This was true even in a patient with previous brain infarcts [25]. Whether other factors contribute to gliosis in the Fabry CNS is unknown. Recent studies suggest that activated astrocytes, marrow-derived fibrocytes and alternatively activated M2 microglia and macrophages contribute to non-Fabry CNS fibrosis [54,55]. These factors should be studied in the context of Fabry disease.

The potential role in fibrosis of additional secondary biochemical processes found in Fabry disease has not been addressed [56]. Compromised energy metabolism has been found both *in vitro* and *in vivo*. Low levels of high-energy phosphate molecules phosphocreatine and adenosine triphosphate (ATP) were observed in Fabry patient hearts and improved with ERT [57]. Parameters of cardiac energy metabolism negatively correlated with progression of Fabry cardiomyopathy [58]. Low glucose utilization was observed locally in 18 brain structures in the alpha-galactosidase A gene knockout mouse [59]. In this regard, low activities of mitochondrial respiratory chain enzymes I, IV, and V were lower in cultured Fabry patient fibroblasts and ATP was marginally reduced [60]. In addition, altered lipid composition of membranes leading to abnormal trafficking and sorting of rafts-associated proteins was observed in fibroblasts [61].

## In search of the mediators of fibrosis in Fabry disease: a potential role for lyso-Gb3

Recently, lyso-Gb3 has been proposed as a promoter of fibrosis in Fabry disease [62]. Having lost a fatty acid, lyso-Gb3 is more water soluble than Gb3 and in some aspects it may behave as an accumulated soluble mediator in a similar manner to glucose and its degradation products that are increased in diabetes. Key differences between lyso-Gb3 and glucose and its degradation products should be recognized; the latter may react with and modify proteins such as type IV collagen in the GBM. In this regard, the fact that some molecular mechanisms of fibrosis may be similar in Fabry disease and DN cannot be construed as a general equivalence of the underlying pathogenesis.

Plasma lyso-Gb3 is dramatically increased in classically affected male Fabry patients, but is also increased in females and is reduced but not normalized following ERT while it is undetectable in normal human plasma [18-20]. These characteristics may contribute to a cross-talk between cells with persistent glycolipid deposits following ERT and may explain observations such a similar mean age at end-stage renal disease for males and for females [63,64]. In this regard, concentrations of lyso-Gb3 observed in plasma of females or ERT-treated Fabry males are biologically active in target cells of Fabry disease in culture even when these cells possess alpha-galactosidase activity [18,62]. Lyso-Gb3 promoted proliferation of vascular smooth muscle cells, but not fibroblasts [18]. In addition, in cultured human podocytes, lyso-Gb3 recruited secondary mediators of inflammation and fibrosis. Thus, in normal human podocytes lyso-Gb3 dose- and time-dependently increased the expression of the fibrogenic cytokine TGF-β1 and increased ECM (fibronectin and type IV collagen) synthesis in a TGF-β1-dependent manner [62]. The fibrogenic response of podocytes to lyso-Gb3 is similar to podocyte responses to a high glucose extracellular milieu [13]. Furthermore, lyso-Gb3 stimulated inflammation similar to high glucose levels does, promoting the expression of the cytokine receptor CD74 [62,65,66]. Lifetime exposure to lysoGb3 correlated with disease manifestations [67]. Plasma lysoGb3 concentration correlated with white matter lesions. In females, plasma lysoGb3 concentration correlated with overall disease severity and LV mass. In addition, lyso-Gb3 reduction on ERT was correlated with LV mass reduction in females and development white matter lesions and stroke [68].

## Clinical consequence of fibrosis and non-invasive assessment of fibrosis in Fabry disease

Imaging techniques in the heart and brain, but not in the kidney can non-invasively assess fibrosis in Fabry disease. In addition, clinical manifestations associated with fibrosis may provide an approximate idea of the extent of underlying fibrosis.

Currently, renal biopsy provides the best assessment of the degree of kidney fibrosis. However, fibrosis may be patchy and the biopsy may not always be fully representative of the whole kidney. Imaging does not yet provide a sensitive assessment and monitoring of kidney fibrosis in humans. Advanced magnetic resonance imaging (MRI) devices allow quantification of renal fibrosis in experimental animals and clinical advances in the field are expected in the near future. MRI or ultrasound identify abnormalities suggestive of kidney fibrosis such as increased echogenicity, decreased cortical thickness and loss of cortico-medullary differentiation in 60% of classically affected males with serum creatinine levels <1.3 mg/dL pointing to kidney fibrosis preceding a decrease in renal function [69]. Urinary protein/creatinine ratio >1g/g, eGFR <45 ml/min/1.73m^2^ or biopsy-proven glomerulosclerosis are associated with progressive kidney disease or a sub-optimal response to ERT [6,70,71], suggesting that these non-invasive biochemical assessments provide insights into both the underlying tissue pathology and response to ERT. Indeed, proteinuria is a known consequence of glomerulosclerosis. However, individual variability and the presence of functional factors that impact on albuminuria and proteinuria or GFR makes unreliable the estimation of the degree of kidney fibrosis from biochemical parameters. Clearly, improved imaging methods or biomarkers are needed to reliably, repetitively and non-invasively assess kidney fibrosis.

**Figure 3:**
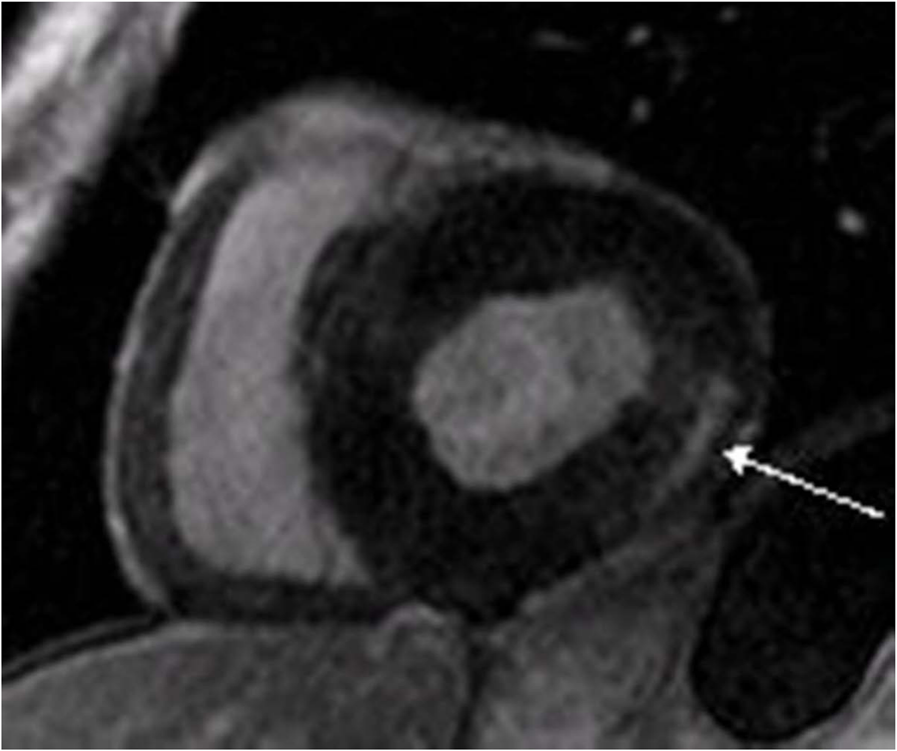
**Cardiac fibrosis.** A magnet resonance imaging short axis view of a 54-year-old male Fabry patient. The arrow indicates the late enhancement positive region of the left ventricle.

Cardiac fibrosis can be visualized either directly using MRI with the late enhancement (LE) technique, or indirectly using functional deformation imaging [32,34] (Figure 3). The gold standard for assessing replacement cardiac fibrosis is late enhancement (LE) imaging during MRI [32,50]. As the distribution of LE positive volume is more localized and not as patchy as in other cardiac diseases it is possible to quantify the LE positive volume in relation to the LV mass with advanced MRI techniques. In a large cohort study, 51% of the Fabry patients (female 44.1%; male 61.9%) showed mid- and trans-myocardial LE with a mean volume of 1.2±1.8% of the LV mass [72]. Female patients with LE positive myocardium presented smaller volumes of LE (0.8±1.3%) than male patients (1.6±2.3%). In a more advanced population even 7.7% of the LV mass was LE positive [32]. In general in both genders, pathological LE is mostly limited to posterior and lateral segments, with different distensions towards the apex but not reaching the apical segments. However, these MRI measurements cannot be performed in all Fabry patients; for patients with end-stage renal disease contrast agents are contraindicated, and MRI examinations cannot be carried out on patients with an implanted cardiac devices or pacemakers [73]. Therefore, other echocardiographic techniques like speckle tracking and strain rate imaging may be useful in these patients for the indirect assessment of replacement fibrosis. Obviously, fibrosis has an impact on myocardial function. When applying tissue Doppler based deformation imaging the strain rate curves extracted from the segment with replacement fibrosis present with a typical double peak sign [74]. Use of this sign allows a qualitative but not a quantitative evaluation for fibrosis of the interrogated segment [73]. As this technique is time consuming and difficult for post processing new imaging techniques like 2D speckle tracking are being developed to assess functional abnormalities. This technique can be applied for the non-invasive evaluation of LE related functional abnormalities in patients with Fabry disease. It is widely available, highly reproducible and easily applicable in most patients. Although a direct quantification of the amount of fibrosis is not possible, a systolic longitudinal strain value >16.5% in the typical postero-lateral region makes replacement fibrosis extremely unlikely [72]. Vice versa, a value <12.5% is very often related to replacement fibrosis and results in the diagnosis of advanced fibrotic stage of the disease. As the evaluation of replacement fibrosis is crucial for staging of the cardiomyopathy, every adult patient should receive a MRI at least once. This is especially important in female patients, because the only sign of cardiomyopathy can be the fibrosis in the postero-lateral wall. Early interstitial fibrosis might have an impact on global systolic and diastolic function but can not directly visualized. MRI based new T1 mapping techniques might be of value for indirect detection of early interstitial fibrosis. However, this is still under investigation. Replacement heart fibrosis may result in heart failure or arrhythmia, including brady-arrhythmias and malignant ventricular arrhythmias [36,75].

**Figure 4:**
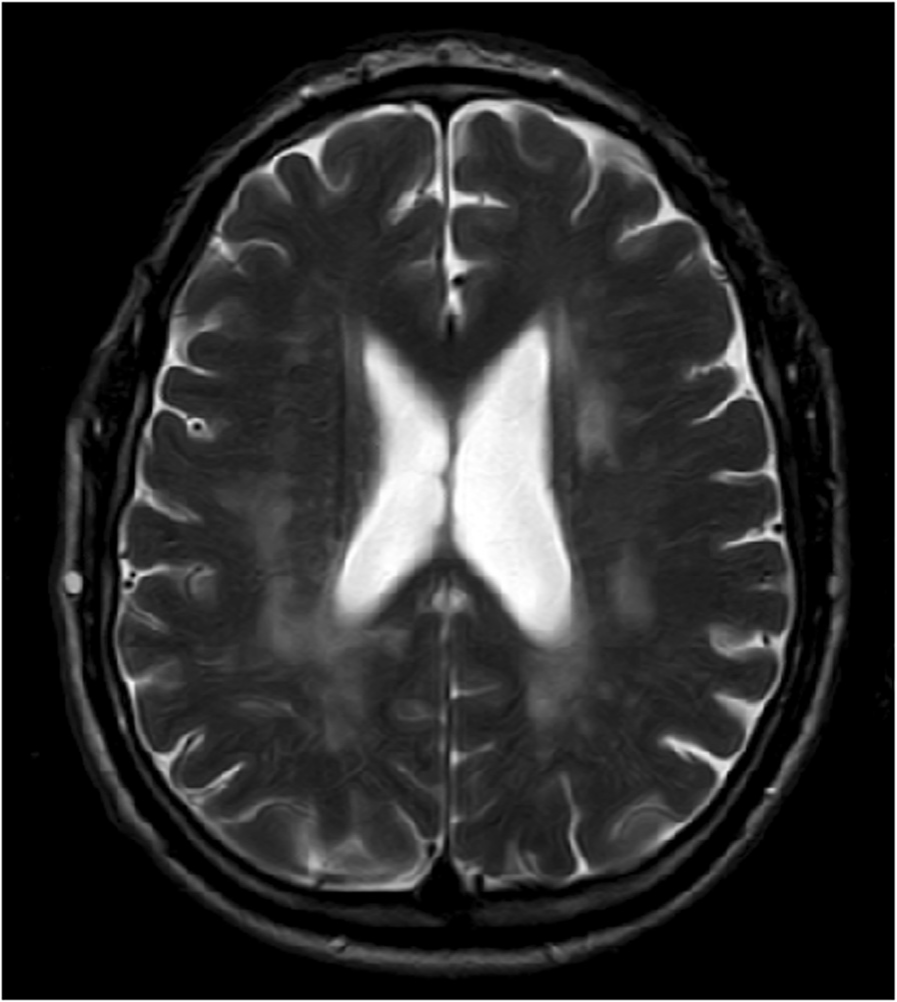
**Central nervous system white matter lesions.** 45-year-old male. T2 Brain MR image showed widespread, punctuated and confluent white matter lesions from periventricular to subcortical spaces. These lesions are associated with severe axonal injury (gliosis).

In the central nervous system white matter lesions (leukoaraiosis) characterized by bilateral and either patchy or diffuse areas of hyperintensity on T2-weighted MRI are a feature of Fabry disease (Figure 4). To what extent they represent gliosis or other features of CNS injury; such as patchy demyelination in Fabry disease is unknown. Studies are needed on the potential relationship to early gliosis of metabolic abnormalities found by proton MR spectroscopic imaging (H-MRSI), Diffusion-Tensor-Imaging (DTI) or 18 fluorodeoxyglucose PET even in the absence of conventional MRI cerebral lesions in Fabry patients [76-81]. Brain levels of N-acetylaspartate (NAA), an aminoacid localized almost exclusively in neurons and axons in mature brain that correlates with axonal density, were lower in Fabry patients than in normal controls when assessed by H-MRSI [76,79]. DTI measures the random translational motion of water molecules as mean diffusivity (MD). Global mean MD values were higher in Fabry patients than in controls [77,80]. 18 fluorodeoxyglucose PET disclosed decreased glucose utilization in some deep and periventricular white matter regions even in the absence of lesions [81]. Politei and Capizzano reported significantly increased mean apparent diffusion coefficient values in posterior paraventricular white matter regions in Fabry patients despite no evident white matter lesions in MRI [78] DTI is useful to predict progression of microvascular injury and eventual development of gliosis in patients at high cardiovascular risk. Stepwise decreases in white matter integrity as measured by both DTI and FLAIR were associated with stepwise increases in white matter lesions risk, emphasizing that these modalities may provide complementary information for understanding the time course of neuronal and axonal degeneration [82]. Additional studies should test whether these results apply to Fabry disease. No obvious relationship was found between MRI white matter lesions and neuropsychiatric symptoms in Fabry patients [83].

## Beyond ERT: management of fibrosis in Fabry disease

The assessment of fibrosis has impact on management and on response to therapy. ERT is the standard therapy for Fabry disease. ERT partially cleared microvascular deposits of GL-3 from the heart, kidney and skin of most Fabry patients [46,84-86]. However, deposits in podocytes may persist for years in adults [6,17]. GL-3 clearance from the myocardium and kidney concurs with a decrease of LV mass and an improvement of regional myocardial function and stabilizes renal function if started early [6,34,74,87,88]. In contrast, ERT may be less effective in the presence of tissue fibrosis identified either by the presence of glomerulosclerosis in renal biopsy, by surrogate markers of kidney fibrosis such as proteinuria >1g/d or an eGFR< 45 ml/min or by evidence of replacement fibrosis in LV [5,6,70,71]. Thus even with ERT, the annual progression of LV replacement fibrosis is 0.7±0.7% in males and 0.2±0.3% in females [89], emphasizing the need to understand the molecular mechanism and optimize anti-fibrotic therapy. This has two clinical implications: a) Before starting with ERT a baseline staging of the extent of fibrosis should be obtained in all patients for adjusting outcome expectations. Disease stabilization is unlikely in the presence of fibrosis. b) Add-on therapies targeting fibrosis may be beneficial in patients with evidence of fibrosis. These add-on therapies are expected to be used in addition to ERT or to any other treatments aimed at correcting the metabolic defect that are developed, such as chaperones or substrate reducing therapy. Ideally clinical trials should address the safety and efficacy of these approaches in Fabry disease. However, clinical trials of these add-on therapies are unlikely given the low frequency of the disease. Until we have clinical trial data, we have to rely on extrapolating concepts that have proven beneficial in other forms of CKD or cardiac fibrosis. In addition, special attention should be paid to elucidating the mechanisms of generation and actions of suspected pro-fibrotic molecules such as lyso-Gb3 as well as in characterizing their receptors, since limiting their production or preventing their pro-fibrotic action might be beneficial in Fabry disease.

The standard of treatment of proteinuric CKD, including DN and FSGS, involves anti-proteinuric therapy with drugs targeting the RAAS such as angiotensin converting enzyme inhibitors (ACEi) or angiotensin receptor blockers (ARBs) [90,91]. Components of the RAAS have direct pro-fibrotic effect that can be demonstrated in cultured cells and animal models in diverse organs that can be prevented by ACEIs, ARBS and anti-aldosterone agents, suggesting a general profibrotic effect of the RAAS beyond specific roles in the biology of specific organs [92,93]. In Fabry nephropathy the combination of ERT and RAAS targeting to decrease proteinuria prevented progression of CKD in patients with baseline estimated GFR <60 ml/min/1.73 m^2^[7]. An ongoing clinical trial is validating this clinical observation (The Fabrazyme® and Arbs and ACE Inhibitor Treatment (FAACET) Study [94]. However, neither study assessed renal fibrosis. Meanwhile RAAS targeting is recommended to lower proteinuria in Fabry disease, in association to ERT [4]. Furthermore, RAAS targeting is also beneficial in chronic cardiomyopathies. Fabry patients with a fibrotic cardiomyopathy generally require comprehensive management of hypertension with angiotensin-converting enzyme inhibitors and ß-adrenergic blocking agents, in addition to ERT [7,95]. A pacemaker implantation might be necessary in cases with symptomatic bradycardia [75]. In addition, patients with late-stage cardiomyopathy who develop life threatening arrhythmias should be evaluated for and eventually provided with insertion of an implantable-cardio-defibrillator (ICD), in addition to pharmacological therapy and ERT [95].

The cell culture observations on the pro-fibrotic role of lyso-Gb3 may have practical therapeutic consequences. In this regard, vitamin D receptor activation with paricalcitol or calcitriol prevented the increase in TGF-β1, CD74 and ECM induced by lyso-Gb3, suggesting that vitamin D receptor (VDR) activation is a potential adjunctive therapy in Fabry nephropathy [62]. A recent clinical trial found inconclusive evidence of an anti-proteinuric effect of the VDR activator paricalcitol in DN [96]. In addition VDR activation has anti-proteinuric and anti-fibrotic effects in a variety of animal models of kidney injury and may also improve LV hypertrophy [97], although the latter was not confirmed in a clinical trial [98]. Guidelines for the general population and CKD patients suggest that vitamin D deficiency should be corrected [91]. Thus, it seems advisable to place particular emphasis in following guidelines on vitamin D management in CKD patients in patients with Fabry disease [5]. Furthermore, specific targeting of molecular mediators of fibrosis such as TGFβ1 is undergoing clinical trials for FSGS [99]. TGFβ1 is a key fibrogenic cytokine [9-11] and was recently found up regulated the enlarged heart of a patient with mucopolysaccharidosis type I (deficiency of α-L-iduronidase) who died from sudden cardiac failure [100]. The mucopolysaccharidoses (MPS) are a group of lysosomal storage disorders (LSD) due to deficiency of enzymes involved in the catabolism of glycosaminoglycans. Like Fabry disease, all types of MPS (particularly MPS-I, II and VI) can present with cardiovascular manifestations, including hypertrophic cardiomyopathy, thickened valvular lesions, and coronary artery lesions. Therefore, it might postulated, and worth testing the hypothesis, that TGF β1 signaling hyperactivity is a pathogenic event common to LSD affecting the heart. Additional mediators of fibrosis undergoing clinical trials for other indications include the Notch system of ligands and receptors [101] and another member of the RAAS, aldosterone [102,103].

A tight control of cardiovascular risk factors, including the use of statins is recommended in Fabry disease. Statins have been reported to have anti-fibrotic activity in kidneys, the vasculature and the heart [104-107]. Together with RAAS targeting, statins have been studied as upstream therapy for atrial fibrillation, that is, the use of non-antiarrhythmic drugs to modify the atrial substrate- or target-specific mechanisms of atrial fibrillation, such as atrial fibrosis, hypertrophy or inflammation [108]. Their potential contribution to the treatment of Fabry fibrosis should be studied.

In conclusion, fibrosis of target organs is an early event in the course of Fabry disease and indicates an impaired response to ERT. A better understanding of the molecular mechanisms of fibrosis may pave the way for the design of add-on therapeutic strategies that improve patient outcomes. Ideally these strategies should be tested in clinical trials.

## Abbreviations

ACEi: Angiotensin converting enzyme inhibitors; A-GAL: Alpha-galactosidase A; ARBs: Angiotensin receptor blockers; ATP: Adenosine triphosphate; CD74: Cluster of differentiation 74; CKD: Chronic kidney disease; CNS: Central nervous system; DN: Diabetic nephropathy; DTI: Diffusion-Tensor-Imaging; ECM: Extracellular matrix; ERT: Enzyme replacement therapy; FAACET: The Fabrazyme® and Arbs and ACE Inhibitor Treatment; FSGS: Focal and segmental glomerular sclerosis; GBM: Glomerular basement membrane; GFR: Glomerular filtration rate; GL-3: Globotriaosylceramide; H_MRSI: MR spectroscopic imaging; LE: Late enhancement; LSD: Lysosomal storage disorders; LV: Left ventricular; Lyso-Gb3: Globotriaosylsphingosine; MD: Mean Diffusivity; MPS: Mucopolysaccharidoses; MRI: Magnetic resonance imaging; NAA: N-acetylaspartate; RAAS: Renin-angiotensin-aldosterone system; TGF-β: Transforming growth factor Beta; VDR: Vitamin D receptor.

## Competing interests

Frank Weidemann, Juan Politei, João-Paulo Oliveira, Christoph Wanner, David G Warnock and Alberto Ortiz are members of the Fabry Registry Boards of Advisors, sponsored by Genzyme. Maria D Sanchez-Niño has received travel money/ speaker fees from Genzyme. David Warnock is a consultant for Genzyme and also has research funding from Genzyme. Alberto Ortiz has received speaking fees from Shire.

## Authors’ contributions

All authors contributed parts of the manuscript according to the medical specialties and reviewed the final version of the text. All authors read and approved the final manuscript.
